# The role of artificial intelligence in diagnostics: A new frontier for laboratory medicine in Africa

**DOI:** 10.4102/ajlm.v14i1.2952

**Published:** 2025-09-26

**Authors:** Talkmore Maruta

**Affiliations:** 1 Department of Programs, African Society for Laboratory Medicine, Addis Ababa, Ethiopia

## Introduction

The past decade has witnessed an unprecedented convergence of data science and healthcare, catalysed by advances in artificial intelligence (AI) and machine learning. Laboratory medicine has not been spared, with AI transforming and improving laboratory diagnostics’ efficiency, accuracy, and speed. The capabilities of AI algorithms, to analyse large data sets, automate tasks, and improve diagnostic accuracy, lead to faster turnaround times, reduced human error, and more personalised patient care. For the African continent, where diagnostic capacity remains uneven and often strained, based on the Lance Report,^1^ the integration of AI presents both a transformative opportunity and a complex challenge. This editorial explores the evolving role of AI in the practice of laboratory medicine and diagnostics, and its potential to close health equity gaps while ensuring ethical, sustainable, and contextually appropriate adoption across Africa.

## Artificial intelligence: An overview in the diagnostic context

Hussein Abbass defines AI simply as ‘the automation of cognition’.^2^ In essence, AI is a science that helps machines find a solution to complex problems in a more humanlike fashion. This generally involves borrowing characteristics from human intelligence and applying them as algorithms in a computer-friendly way. In diagnostics, AI systems are trained on large data sets to identify patterns in clinical, molecular, or laboratory data. It has found its application in molecular and genomic diagnostics, laboratory-based diagnostics, for example interpretation of microscopy, haematology, or clinical chemistry results, point-of-care and wearable diagnostics, and clinical decision support systems. For Africa, where the ratio of diagnostic professionals to patients is significantly lower than global averages, AI can help fill critical human resource and infrastructure gaps, support task-shifting, and accelerate access to timely and accurate diagnoses.^3^

## The role of artificial intelligence in diagnostics in Africa

### Addressing diagnostic delays and errors

One of the most persistent barriers to effective healthcare in Africa is delayed or missed diagnosis. Artificial intelligence-enabled diagnostic tools can assist in rapidly analysing laboratory results, flagging abnormalities, and suggesting possible diagnoses. Automated digital pathology platforms can analyse tissue biopsies or blood smears with speed and precision, potentially reducing diagnostic turnaround times from days to minutes.

### Enhancing diagnostic access in underserved regions

With 47% of the global population having little to no access to diagnostics, particularly in Africa’s rural or resource-limited areas, access to trained laboratory professionals or specialised services, such as pathologists, may be non-existent. Artificial intelligence-powered mobile platforms and point-of-care devices can extend diagnostic capabilities to the frontlines, allowing community health workers to leverage advanced tools for screening diseases such as tuberculosis, malaria, or cervical cancer.

### Supporting laboratory workflow efficiency

Workflow is central to efficiency and incorporation of AI into laboratory processes improves efficiency in workflow, quality control, inventory management, and test interpretation. McKinsey and Harvard reported that AI can save the healthcare industry US$360 billion a year, because of improved efficiencies.^4^ Automated image analysis for haematology (e.g. white blood cell classification), microbiology (e.g. culture plate reading), and clinical chemistry (e.g. flagging abnormal trends) can alleviate the workload of laboratory professionals and improve consistency in result interpretation.

### Integrating multimodal data for complex diagnoses

Complex diseases, such as cancer or antimicrobial resistance, require integration of clinical, laboratory, imaging, and molecular data. Artificial intelligence systems are uniquely suited to synthesise these diverse data streams to support differential diagnoses and personalised treatment decisions.

**FIGURE 1:**
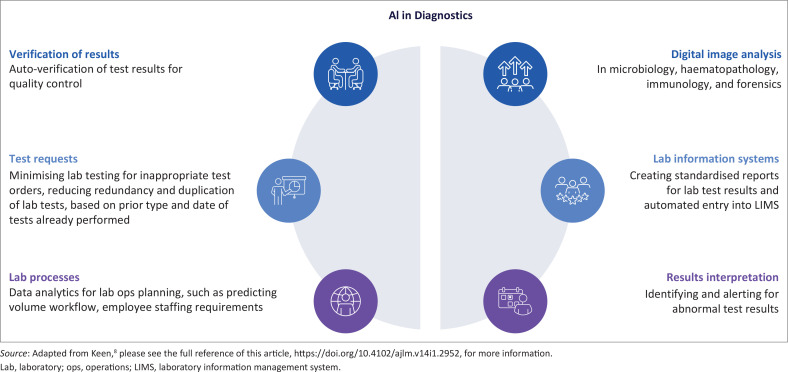
The role of artificial intelligence in diagnostics.

Many countries in Africa have begun to realise the advantages of AI in diagnostics. South Africa and Zambia are using computer-aided detection of tuberculosis using chest X-rays and algorithms such as CAD4TB,^5^ automated malaria detection from Giemsa-stained blood films is under evaluation in Kenya and Ghana, aiming to improve diagnostic accuracy and reduce inter-observer variability,^6^ and predictive algorithms for antimicrobial resistance patterns, using laboratory surveillance data, can assist in early identification of resistance trends and guide empirical therapy.^7^ In addition to these specific examples, AI has been incorporated into Laboratory Information Management Systems to automate interpretation and generation of laboratory reports. Modules can also be built into the equipment test menus for reagents stock management and staff productivity. These examples underscore the continent’s growing engagement with AI in diagnostics, although they remain largely pilot-driven and limited in scale. Figure 1 highlights the role of AI in diagnostics.

## Challenges and risks in artificial intelligence-enabled diagnostics

While the benefits of AI in diagnostics are substantial, several challenges must be addressed to ensure equitable, effective, and responsible deployment in Africa.

### Data quality and representativeness

Artificial intelligence algorithms are only as good as the data used to train them. Most AI diagnostic models are developed using data sets from high-income countries, which may not generalise to African populations because of differences in disease prevalence, comorbidities, and health system contexts. There is an urgent need to generate and curate high-quality, diverse, and representative data sets from African settings to train contextually relevant models.

### Infrastructure and digital divide

Reliable electricity, internet connectivity, and computational infrastructure are preconditions for implementing AI tools. Many laboratories and health facilities across Africa lack these basics. Without parallel investments in infrastructure, AI technologies risk exacerbating existing health disparities.

### Ethical and regulatory concerns

Artificial intelligence systems raise complex ethical questions around data privacy, consent, accountability, and transparency. In the diagnostic context, who is responsible when an AI system makes a wrong diagnosis? What safeguards exist to prevent bias in decision-making? African governments must urgently develop regulatory frameworks to govern AI in healthcare, drawing on global best practices but tailored to local contexts.

### Capacity building and workforce integration

The adoption of AI in diagnostics requires a workforce that is not only AI-aware but AI-capable. Laboratory scientists, pathologists, and clinicians must be trained to understand how AI works, how to critically interpret its outputs, and how to integrate it into clinical workflows. Additionally, new roles, such as health data scientists and clinical AI specialists, must be cultivated within Africa’s health education systems.

### Job replacements

Although specialised skills such as laboratory scientist and pathologist are still below set global standards, most African countries are still battling with very high unemployment levels. From that angle, AI maybe seen as an option that will result in job replacements for those few that are currently employed.

## Ethical and equity considerations for African contexts

Artificial intelligence holds the potential to democratise access to diagnostics, but only if its development and deployment are guided by principles of equity, inclusion, and justice. Africa must avoid becoming merely a consumer of externally developed technologies. Instead, investments must be directed towards homegrown innovation ecosystems, enabling African researchers, institutions, and entrepreneurs to co-create AI solutions that reflect the continent’s diverse needs.

Artificial intelligence systems must be transparent and explainable. The ‘black box’ nature of AI – wherein users may not understand how decisions are made – can undermine trust in diagnostic processes. Emphasis must be placed on developing explainable AI models, especially in life-critical scenarios.

Artificial intelligence is rapidly reshaping the landscape of diagnostics, offering unprecedented opportunities to enhance laboratory medicine in Africa. From improving accuracy and turnaround times to extending diagnostic reach in underserved regions, AI holds immense promise for addressing the continent’s persistent health system gaps. However, realising this potential requires more than just technological adoption – it demands intentional investment in infrastructure, ethical frameworks, local innovation, and capacity building. As African countries navigate this transformative frontier, a people-centred, inclusive, and context-aware approach will be essential to ensure that AI serves as a catalyst for equitable, high-quality healthcare rather than a source of further disparity. The future of diagnostics in Africa lies not just in smarter machines, but in smarter systems that empower professionals, protect patients, and promote health for all.
